# Wrinkling Analysis and Process Optimization of the Hydroforming Processes of Uncured Fiber Metal Laminates for Aircraft Fairing Structures

**DOI:** 10.3390/polym17162267

**Published:** 2025-08-21

**Authors:** Yunlong Chen, Shichen Liu

**Affiliations:** School of Aerospace Engineering, Xiamen University, Xiamen 361105, China

**Keywords:** fiber metal laminates, hydroforming, aircraft fairings, wrinkling, finite element modeling

## Abstract

Lightweight composite structures like fiber metal laminates (FMLs) are widely used to manufacture aircraft structures and substitute metallic parts. While the superior mechanical performance of FMLs, including their high specific strength and excellent impact and fatigue resistance, has gained the interest of many researchers in the aerospace manufacturing industry, there are still some challenges that need to be considered. Conventional approaches like lay-up techniques and autoclave molding can achieve the relatively simple FML parts with large radii and profiles required for aircraft fuselages and flat skins. However, these methods are not suitable for forming complex-shaped structural parts due to the limited failure strain of fiber-reinforced materials and complex failure modes of the laminates. This research puts forward a new methodology that combines the hydroforming and subsequent curing process to investigate the feasibility of manufacturing complex aircraft parts like fairings made by FMLs. In this research, wrinkle formations are analyzed under various parameters during the hydroforming process. The geometrical shape of the initial blanks and the parameters, including blank holder force and cavity pressure, have been optimized to avoid flange edge wrinkles, and the addition of local support materials contributes to improving local wrinkling in the sharp corners. A finite element model (FEM) taking material laws, interlayer contacts, and boundary conditions into account is used to predict the dynamic hydroforming process of the fiber metal laminate, and experimental works are carried out for its verification. It is expected that the proposed method will reduce both costs and time, as well as reducing laminate defects. Thus, this method offers great potential for future applications related to manufacturing complex-shaped aerospace parts.

## 1. Introduction

Aircraft fairings are structures whose primary function is to produce a smooth outline and reduce drag force during flight. These structures are employed to protect systems and their underlying components from adverse external environmental factors. The traditional use of aluminum alloys on aircraft fairings can improve appearance and achieve aerodynamic benefits such as high strength and stiffness. The application of composite materials such as fiberglass-reinforced polymer (GFRP) and carbon-fiber-reinforced polymer (CFRP) has grown dramatically over the past decade, resulting in greater fuel efficiency and lower costs in the aerospace industry.

Fiber metal laminates are lightweight composites made using alternating strata of fiber-reinforced composite layers and metal alloy sheets bonded together. These hybrid structures can be utilized for different requirements using various material constituents, which present superior results compared to the simple sum of their separate effects; moreover, they demonstrate excellent performance in fatigue and corrosion resistance, and show superior mechanical characteristics and damage tolerance [1,2,3]. For fiber metal laminates based on thermoset polymers, like glass-laminate-aluminum-reinforced epoxy (GLARE), the matrix depends on the chemical reactions of polymer constituents to develop its functionality during the curing process. This typical cross-linking mechanism, which solidifies the epoxy resins, is irreversible and usually performed in special autoclaves that are capable of maintaining high temperatures and pressures [4,5,6,7].

Traditionally, there are two main approaches utilized for the manufacturing process of thermoset-based fiber metal laminates. One of the common methods is based on automatic or manual lay-up techniques, where the pre-consolidated laminates are covered in a vacuum bag and cured in an autoclave. This method proves to be more feasible for the manufacture of simple monolithic structures such as aircraft fuselages and flat skins [8]. Utilizing lay-up and autoclave processes can result in better-quality final products; however, some limitations, like lengthy production time and high operational prices, make its use challenging, especially for small- and medium-sized structures in mass–volume applications [9,10]. The alternative method proposed is to apply a sheet-metal-forming technique to manufacture relatively small and complex-shaped structures. In this method, structures are created by forming multilayer metal sheets and stamping or deep drawing the desired shape in a single step. Then, a curing agent and hardener are prepared and applied to dry woven fabric using heat and pressure [11,12], or through the vacuum-assisted resin transfer molding (VaRTM) process [13,14]. The application of these processes is cost-effective due to the replacement of high-cost pre-impregnated materials with lower-cost dry fabric reinforcements. However, the uneven resin flow and distribution during consolidation, especially in areas with a smaller radius and in critical regions, can negatively impact the final quality of the product.

In order to minimize adverse influences on the formability of fiber metal laminates due to the limited failure strain of fiber-reinforced composites, a feasible technique is proposed, which uses uncured fiber metal laminates rather than cured one- or multilayer metal sheets to manufacture FML products [15,16,17,18]. Hydroforming processes, which use liquid oils to replace rigid tools, are applied first to increase the forming limit and layer compactness. Then, the final part can be obtained by vacuum solidification of the hydro-formed part. This process is beneficial in suppressing the buckling phenomenon attributed to the instability of the thin laminates, and improves the strength and stiffness of the final product. In previous research, the effects of fiber orientations and fiber types have been investigated to validate the feasibility of forming fiber metal laminate parts [19,20]. The results indicate that the glass-fiber-reinforced polymer with quasi-isotropic (0°/90°/45°/−45°) lay-up for fiber metal laminates shows better formability under various conditions. However, the wrinkling defect observed during the hydroforming process still occurs and needs to be studied.

Wrinkling defects are one of the most prevalent material instabilities that occur in sheet metals that are formed by stamping or deep drawing processes. This phenomenon usually occurs in the flange or free-forming (unsupported) zone due to internal compressive stresses. When using hydroforming as a manufacturing method, the sheet is supported by a bed of pressurized viscous fluid, which provides a through-thickness compressive stress that delays the onset of tensile instabilities and reduces the formation of wrinkles due to tensile frictional forces [21]. To inhibit wrinkling in the flange areas, blank holders must apply normal stresses at the blank surface, while the fluid cavity should be uniformly loaded to fill the desired shapes [22,23]. Besides wrinkles in metal sheets, fiber wrinkling is also a critical defect pattern for fiber metal laminate, particularly in the corner regions. Kenan and Nuri [24] found that the number of wrinkles increases when prepregs are first stacked on a flat plate and then bent to conform to a corner area surface, compared to the conventional lay-up procedure. These wrinkles negatively affect the strength of the parts and influence the deformation behavior after curing. Some researchers have demonstrated that fiber wrinkling occurs when local shear builds up if the fabric continues to be deformed beyond the locking angle. Other studies suggest that the wrinkle formation may result from fiber overlap in woven fabric caused by in-plane compressive forces [25,26].

Based on previous research, the principles governing wrinkle formation during the hydroforming process of fiber metal laminates remain insufficiently understood. In order to manufacture relatively complex parts with improved quality, a middle-sized aircraft fairing is employed to investigate forming behavior and analyze wrinkle formation using uncured fiber metal laminates. In this research, numerical simulations and experimental tests are conducted to examine the influence of hydroforming process parameters on the wrinkling length in the outer flange areas. Additionally, local wrinkling defects in sharp corner regions are explained and optimized through the use of local support materials. This research demonstrates significant potential for future applications in manufacturing complex-shaped aerospace structures.

## 2. Materials and Methods

### 2.1. Materials

**Figure 1:**
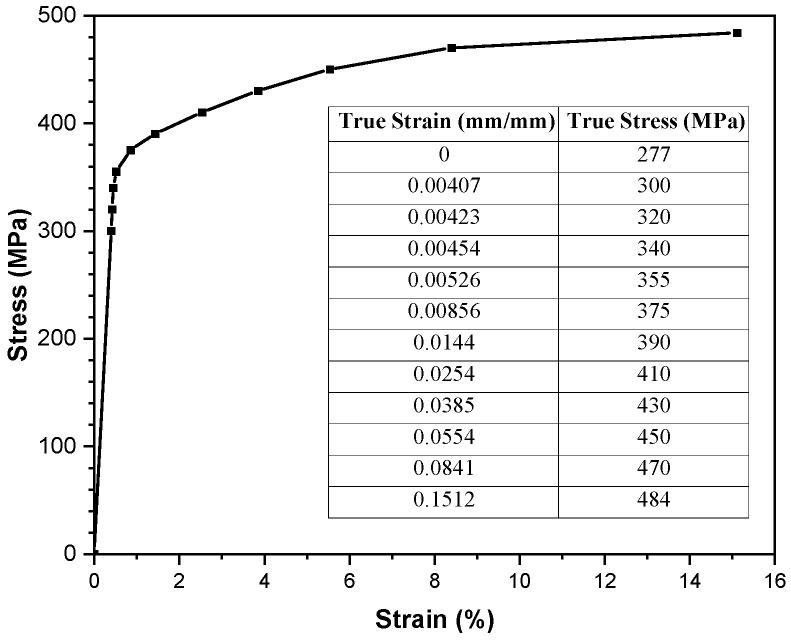
Stress–strain curve for aluminum alloy sheet 2024-T3 [27,28].

Glass-laminate-aluminum-reinforced epoxy (GLARE) is the most common type of fiber metal laminate applied in aircraft structures. The skin layers use aluminum alloy (Al) 2024-T3 with a thickness of 0.3 mm, and the core materials are woven glass fabric prepreg WP 9011 manufactured by Guangwei Composite Company, Weihai, China. The woven prepreg is a plain weave E-glass fabric with a nominal thickness of 0.2 mm; the fabric reinforcement is pre-impregnated with a thermosetting epoxy system. Laminate samples are prepared using a pre-cured treatment where Al 2024-T3 is washed with alkaline and anodized with phosphoric acid for better adhesive bonding between the aluminum and prepreg layers. After that, the assembly is placed in a vacuum bag with 1 bar pressure for 8 h at room temperature to eliminate any voids and bubbles produced between interlayers. During the manual lay-up process, the warp orientations of all woven prepregs are aligned with the rolling direction of the aluminum alloy 2024-T3 sheet. The tensile properties of Al 2024-T3 are tested at room temperature based on the ASTM E8 Standard, while the uncured prepreg tests comply with the ASTM D-3039 Standard [27,28]. The mechanical properties of the aluminum sheet for the laminate are shown in Figure 1; the lay-up configurations from this research are presented in Table 1.

### 2.2. Structures

**Figure 2:**
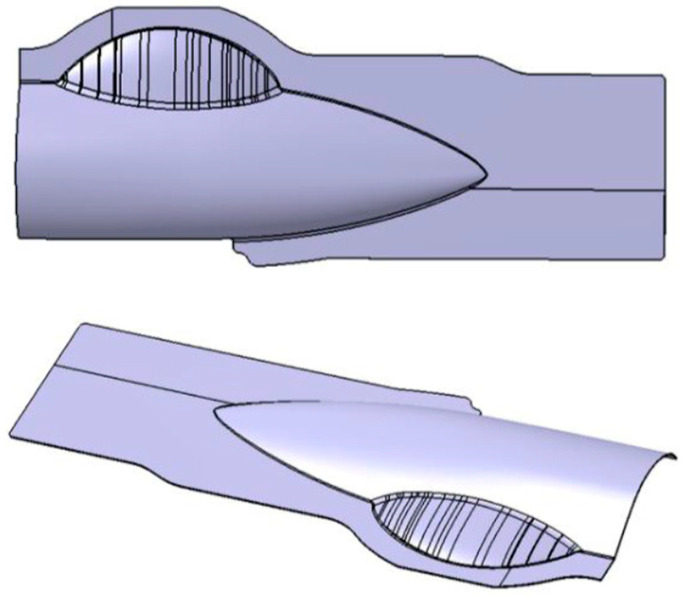
Geometrical shape of the aircraft fairing.

**Figure 3:**
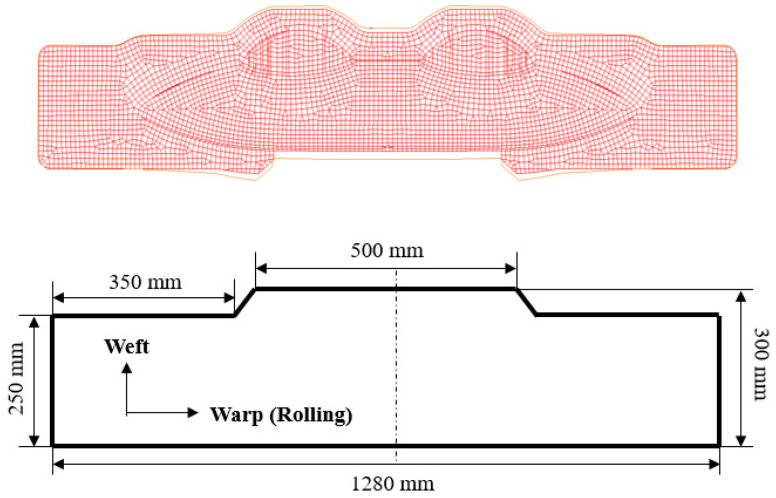
Initial blank optimization by reverse billet method.

The geometrical shape of the aircraft fairing structure is shown in Figure 2. The length and width of the part are 600 mm and 260 mm, respectively. The main cavity incorporating a small cavity can be clearly observed from the figure, and the depths of the two cavities are measured to be 36.5 mm and 23 mm. The fillet radius between the flange and the deep cavity area is designed to be 3.8 mm. Due to the shape characteristics of the target fairing structure, a two-pieces-in-one-mold method is applied. The shape of the initial blanks can be optimized through the reverse billet engineering function from sheet-metal-forming software LS-DYNA R9.3.1 (ANSYS, Pittsburgh, PA, USA) [29]. Based on the reverse billet method and easy-machining principle, the shape and dimensions are simplified and designed as shown in Figure 3.

### 2.3. Hydroforming

**Figure 4:**
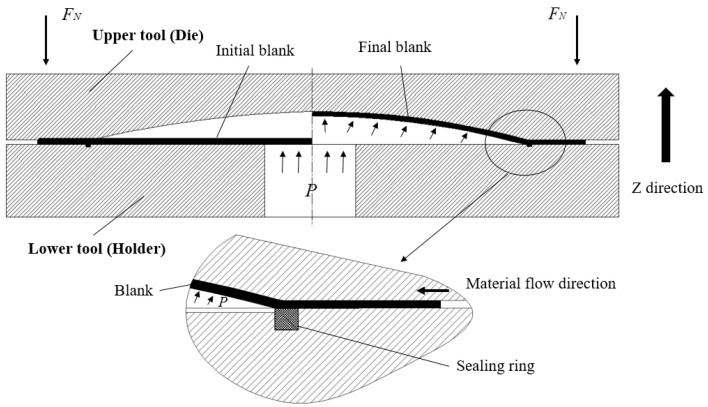
Schematic diagram of the hydroforming system of the aircraft fairing part.

The Glare laminate hydroforming tests for aircraft fairing parts were performed using a 500-ton hydroforming machine from Chengdu Aircraft Industrial Group, Chengdu, China. The machine is controlled by a hydraulic feed system, where all forming process parameters, such as blank holder force and gap, tool feed rate, and cavity pressure, can be adjusted and monitored. A schematic diagram of the fairing hydroforming system is shown in Figure 4. As presented in the figure, the flange area of the part is formed by a rigid blank holder after the entire system is closed, while the deep cavity region is formed through active liquid filling. The liquid sealing device is used to seal the gap between the material in the flange area of the part and the upper surface of the blank holder, preventing pressure loss due to liquid overflow from the main inlet. After the upper tool moves in the *Z*-axis direction for mold closing, the cavity liquid acts on the initial blank for deforming the desired shape of the fairing part.

During the hydroforming process, blank holder force and cavity pressure are the two most critical factors influencing the formability of the fairing parts. Here, a single layer of 0.3 mm thickness aluminum sheet was initially formed under various conditions to explore the feasibility of achieving good formation results. The use of a single aluminum sheet can not only investigate a possible range of forming parameters to reduce the number of tests and examine the impact of process variables, but also serve as a reference sample to analyze the influence of fiber-reinforced composites on the formation of certain defects. The design of the experimental parameters is presented in Table 2, and the effect of the blank holder force and cavity pressure on the wrinkling of Glare layers is discussed in detail in Section 3.2.

### 2.4. Numerical Simulations

Finite element analysis software Abaqus/Explicit 6.14 (Dassault, Paris, France) was utilized to simulate the Glare laminate hydroforming process of the fairing part and analyze the defect generation in multilayer sheets. The model consists of a blank holder, a die, and two different material layers for the laminate. The blank holder and die were modeled as four-node 3D bilinear rigid quadrilateral elements with a mesh size of 2 mm, while the laminate blank was treated as a deformable body using S4R elements (four-node doubly curved shell elements with reduced integration) with the same mesh size. A mesoscopic approach was applied, which divides the laminate into independent layers of homogeneous metal sheet and anisotropic fabric prepreg. Orthotropic elastic material with two-dimensional Hashin damage criteria was considered for the prepreg layers, while isotropic behavior with elastic–plastic properties was applied for the metal sheet layers. Additionally, the boundary conditions for both the upper and lower tools, as well as the laminate, play an important role in defect and damage response. Friction contact interfaces were used to simulate the interactions between the mold and blank layers, with a friction coefficient assumed to be 0.1. Interface cohesive elements between the metal sheet layer and the fiber prepreg layer were applied to simulate the laminate deformation behavior, with performance defined by penalty stiffness, initiation strength, and critical fracture toughness, as shown in Table 3. Wrinkling defects can be clearly observed and measured by the wrinkle lengths following the formation process in the simulation models. Comparisons between the experimental and simulation results are presented in order to verify model accuracy and help optimize better-formed fairing parts.

## 3. Results and Discussion

### 3.1. Part Wrinkling Analysis

**Figure 5:**
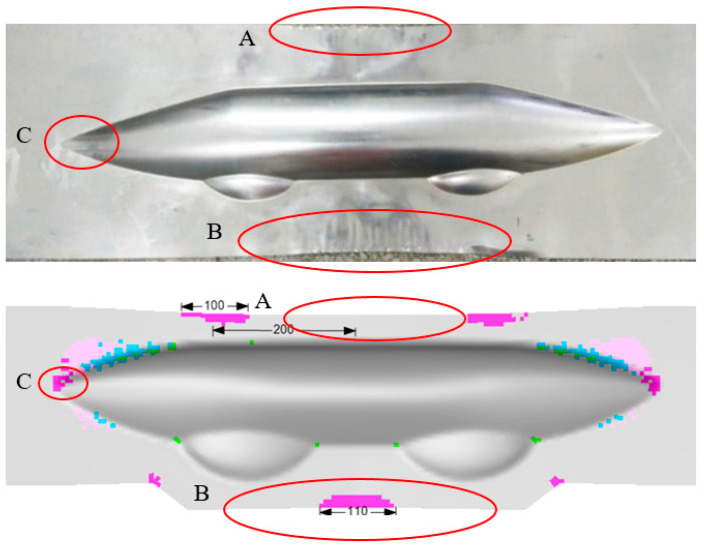
Experimental and numerical results of the 0.3 mm aluminum sheet hydroforming (A—top flange region, B—bottom flange region, C—sharp corner region, red circle-wrinkle accumulation and different colors indicate the degree of wrinkling).

The hydroforming process begins by investigating the formability of the 0.3 mm aluminum sheet on the machine. As shown in Figure 5, three potential wrinkling defect areas are identified, with region B exhibiting the widest wrinkling length of 110 mm, while wrinkling defects are scarcely observed in the outer flange area of the sharp corner (region C). The primary cause of these defects stems from the minimal thickness of the aluminum plate. The 0.3 mm sheet is more prone to developing unstable wrinkles, even under optimal process parameters. The results indicate that a high-quality part can be formed at a maximum cavity pressure of 20 MPa, while blank holder forces ranging from 1500 kN to 2500 kN show minimal influence on defect formation and wrinkling length in single aluminum blanks. The wrinkles can be removed using a cutting tool to perserve the primary shape of the fairing part.

**Figure 6:**
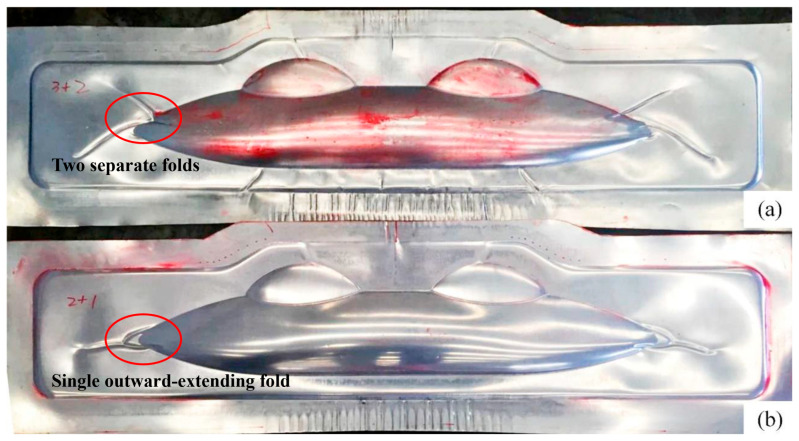
Experimental results of the Glare hydroforming process at 1500 kN and 20 MPa: (**a**) Glare 3/2; (**b**) Glare 2/1.

**Figure 7:**
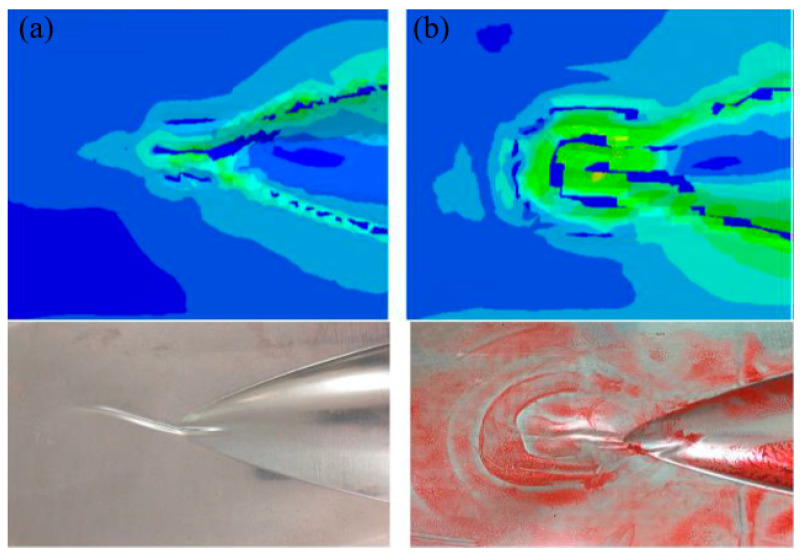
Comparison between the simulation and experimental results of wrinkle formation in region C: (**a**) Glare 2/1; (**b**) Glare 3/2.

After investigating the forming behavior of the single aluminum sheet, multilayer blanks of Glare 2/1 and 3/2 were used for the hydroforming process under identical conditions. As evident in Figure 6, unlike the single aluminum sheet which undergoes slight wrinkling in the top and bottom flange areas, different lay-ups of Glare laminates demonstrate severe wrinkling in the same regions. In addition, as the number of material layers in the laminate increases, both the wrinkling lengths and defect severity become more pronounced. This phenomenon results from the increasing laminate thicknesses with additional layers, along with the redistribution of compressive stress and strain in the aluminum layers due to the inclusion of fiber prepregs in the middle layer. Since fiber materials primarily exhibit tensile deformation behavior when forming, wrinkling manifests due to prepreg overlap while the aluminum sheet undergoes compressive strain at corresponding locations. In the outer flange of the sharp corner area (as marked in the figure), wrinkles develop in two distinct patterns for different lay-ups. The Glare 2/1 structure forms a single outward-extending fold, while the Glare 3/2 structure produces two separate folds. A comparison between the simulation and experimental results for these different lay-ups in the sharp corner region is presented in Figure 7. For the Glare 2/1 and 3/2 laminates used in this study, the netrual layers correspond to the fiber prepreg and aluminum sheet layers, respectively. During the hydroforming process, the upper portion of the netrual layer undergoes tensile stress while the lower portion experiences compressive stress. In addition to wrinkles in compressed fiber prepreg layers, portions of the middle aluminum layer also develop wrinkles, leading to these distinct wrinkling behaviors.

**Figure 8:**
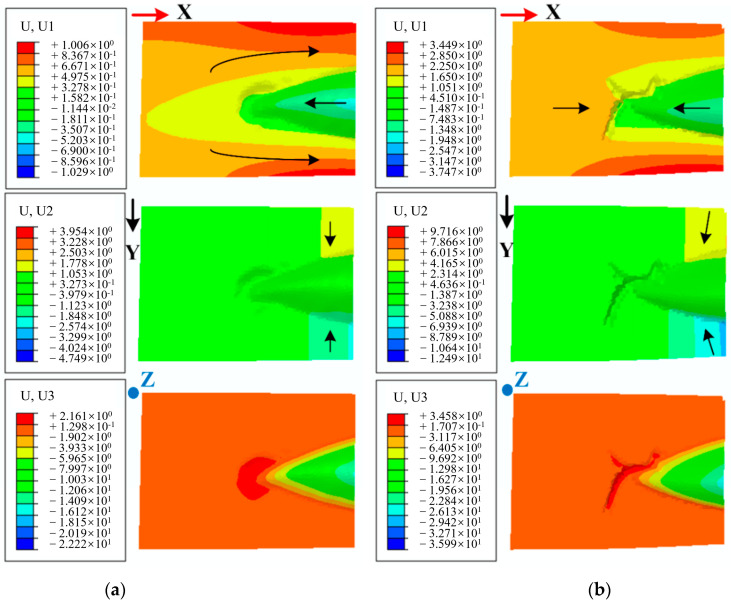
Displacement distribution of the sharp corner region for the fiber prepreg layer. (**a**) Forming depths: 23 mm. (**b**) Forming depths: 36.5 mm.

The wrinkling phenomenon in the top and bottom flange areas of the laminate (region A and B in Figure 5) results from the combined effects of uneven material flow in the sheet and the inherent instability of thin aluminum plates, which is essentially identical to the deformation behavior observed in single aluminum plates. This type of laminate wrinkling can be mitigated through the optimization of process parameters such as blank holder force and cavity pressure. However, the wrinkles in the sharp corner area (region C in Figure 5) primarily stem from the specific geometry, material anisotropy, and inconsistent deformability within the laminate. Figure 8 illustrates the displacement distribution as an indicator of material flow in the fiber prepreg layer of the Glare 2/1 laminate within the sharp corner region. U1, U2, and U3 represent displacements in the X, Y, and Z directions of the globe coordinate system, respectively. When the two small cavities reach their forming depth of 23 mm, deformation in the X direction remains minimal, and material flow into the deep cavity area proves difficult. In addition, material on both sides of the sharp corner flows in the +X direction, while material inside the cavity flows in the opposite –X direction, inevitably causing accumulation and bulging in this region. This results in material flow in the Z direction and increased flows along the Y direction as the cavity width expands. When reaching the maximum depth of 36.5 mm, clear observation reveals that accumulated material protruding outside the sharp corners is compressed into distinct wrinkle defects. This demonstrates that wrinkles in region C originate from material accumulation during hydroforming; these are attributable to the poor compression stability and deformability of both the thin aluminum sheet and fiber-reinforced prepreg. Consequently, this type of wrinkle defect proves difficult to eliminate and shows minimal response to adjustment in the hydroforming process parameters. In Section 3.3, a local support optimization method will be introduced to address these wrinkle defects and produce high-quality fairing parts that are suitable for production.

### 3.2. Process Parameter Optimization

**Figure 9:**
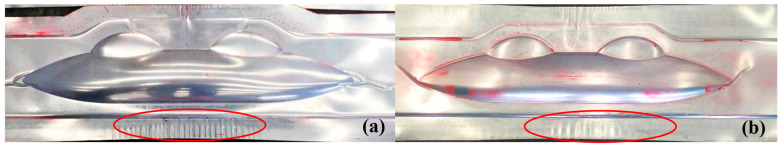
Experimental results of Glare 2/1 laminate under different blank holder forces. (**a**) FBH=1500 kN. (**b**) FBH=2000 kN.

**Figure 10:**
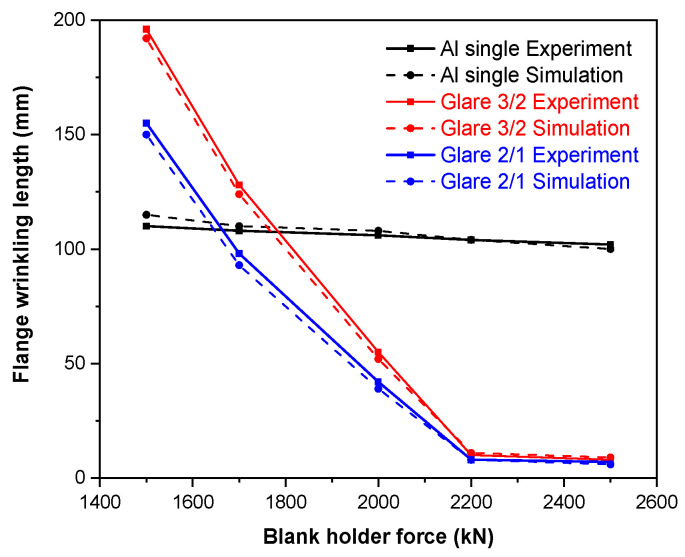
The flange wrinkling length with different blank holder forces at 20 MPa cavity pressure.

Based on the mechanisms of wrinkle formation discussed previously, optimization of the hydroforming process parameters presents an effective solution for controlling wrinkles and achieving high-quality formation of the fairing part composed of fiber metal laminates. The initial experimental results for Glare 2/1 laminate hydroforming under different blank holder forces are shown in Figure 9. Significant improvements in wrinkling defects are shown in the top and bottom flange regions with the increase in blank holder force, though the wrinkles in sharp corner areas remain unaffected. This phenomenon can be attributed to the material flow mechanisms of the laminate. In the flange regions, material flows for both aluminum sheets and fiber prepregs primarily occur along the width direction. Wrinkling develops due to width reduction in the central flange areas, with more pronounced wrinkles observed on the deep cavity side. Increasing force application on the flange area restricts material flow into cavities, efficiently controlling the wrinkles by minimizing width reduction. Consequently, numerical and experimental approaches employing flange wrinkling length (highlighted by red circles in Figure 9) as a key parameter are combined in order to optimize the process parameters for controlling wrinkling in the flange. Figure 10 illustrates that flange wrinkling length gradually decreases with the increase in blank holder force at a cavity pressure of 20 MPa for Glare materials, while remaining nearly constant for single aluminum sheets. When the blank holder force exceeds 2200 kN for both Glare 3/2 and Glare 2/1 laminates, wrinkling length stabilizes at 10 mm and 8 mm, respectively.

**Figure 11:**
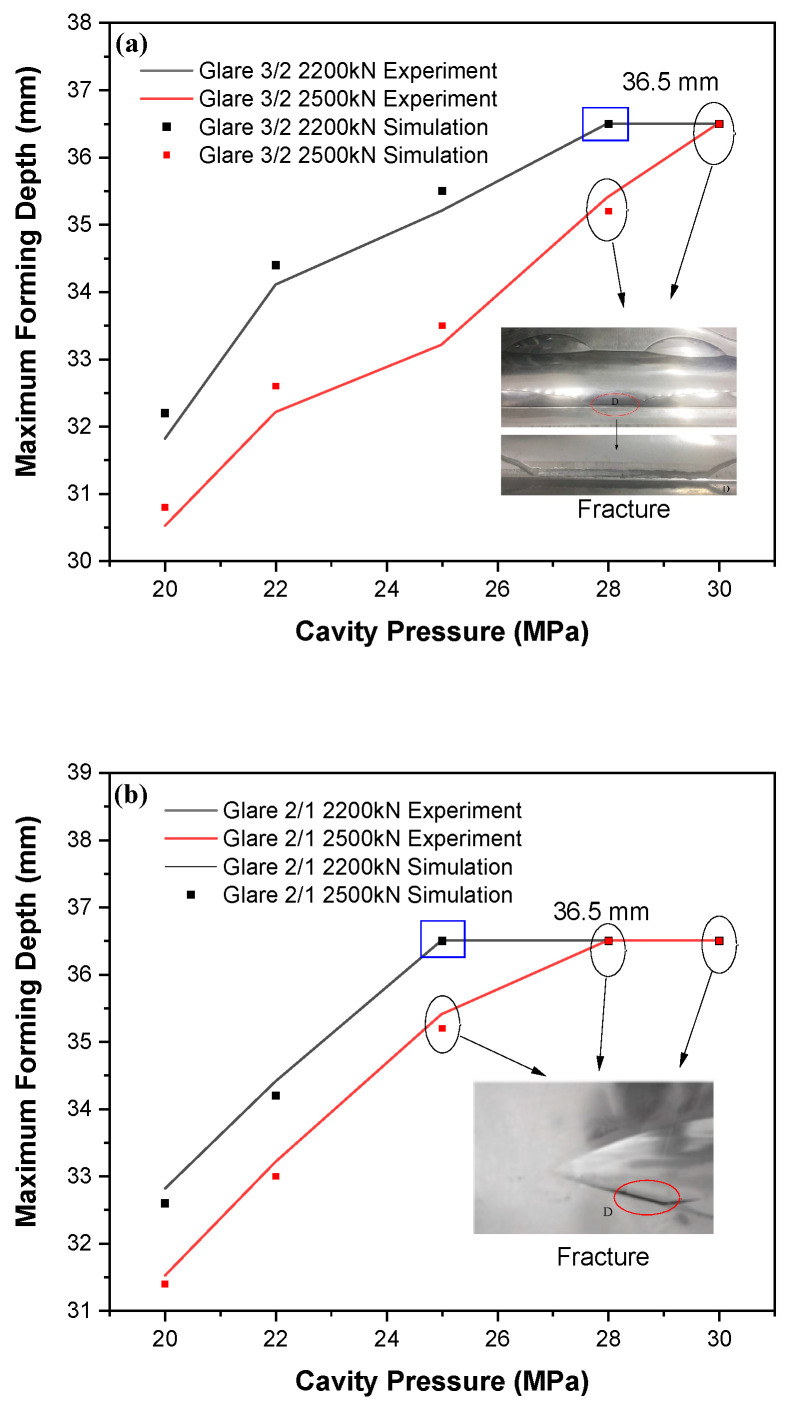
The maximum formation depth at different cavity pressures for Glare laminates: (**a**) Glare 3/2 laminates; (**b**) Glare 2/1 laminates.

Although increasing the force can reduce wrinkles, a higher cavity pressure is necessary to fully fill the cavity shape. However, excessive pressure may cause laminate fracture due to severe reductions in wall thickness from material overstretching. Therefore, we selected blank holder forces of 2200 kN and 2500 kN, where the flange wrinkling length reaches its minimum, and further measured the forming depths at cavity pressures exceeding 20 MPa. The maximum forming depths measured for different Glare materials are presented in Figure 11**.** For Glare 3/2 laminates, the optimal pressure is 28 MPa at 2200 kN, enabling complete mold filling. When reducing the lay-up for Glare 2/1, the fracture initiates slightly earlier, requiring a lower pressure of 25 MPa under the same force as for the higher-quality parts. The figure demonstrates that increasing the cavity pressure or blank holder force beyond these values results in either incomplete filling or cavity fracture. Notably, fractures in Glare 3/2 originate at the central cavity position and propagate outward, while the failure location for Glare 2/1 happens near the sharp corner areas. Additionally, a slight reduction in wrinkling length occurs at optimized pressures, with the formation of only minor wrinkles that are removable during the post-curing process.

**Figure 12:**
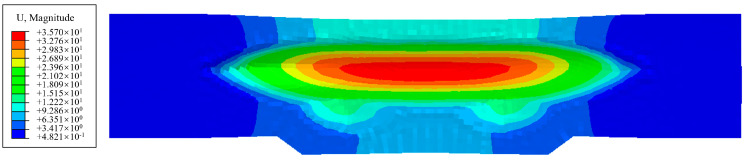
Simulation results of the displacement and material flow diagram for the fiber prepreg.

The material flow in the sharp corner area of the fairing part exhibits distinct characteristics, primarily occurring along the longitudinal direction with minimal displacement, as shown in Figure 12. This observation leads to the conclusion that wrinkle formation mainly results from fiber accumulation and overlap within the laminate under compressive stresses. Adjusting the process parameters in hydroforming has little influence on wrinkle control in the sharp corner areas of fairing parts. These specific wrinkles can only be effectively controlled by altering the stress state to tension, especially for the fiber prepregs. Consequently, a local support optimization method has been developed to control the wrinkles on the sharp corners in order to improve the formation quality of the fairing part.

### 3.3. Local Support Optimization

**Figure 13:**
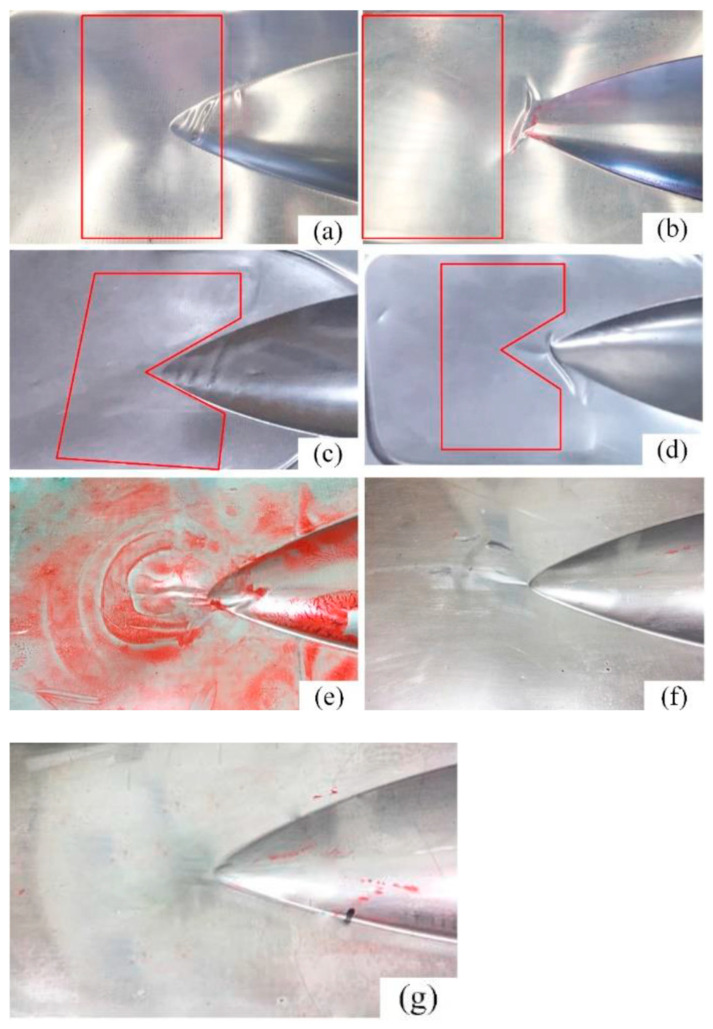
Experimental results wrinkling in the sharp corner areas using different local support methods: (**a**) 10 mm square steel plate 1; (**b**) 10 mm square steel plate 2; (**c**) 10 mm conformal steel plate 1; (**d**) 10 mm conformal steel plate 2; (**e**) 5 mm conformal polyurethane rubber with 5 mm local steel plate; (**f**) 5 mm conformal polyurethane rubber with 5 mm local polyurethane rubber; (**g**) 1 mm conformal steel plate with 9 mm local polyurethane rubber.

Due to the unique geometry of the fairing part and the distinctive material characteristics of the laminate, wrinkle defects in the sharp corner regions need to be improved through a localized constraint method. This section investigates different local support configurations and their positional arrangement in order to optimize material deformation and control wrinkling in fiber metal laminates. The experimental setup compares seven distinct local support approaches against an unsupported reference, as detailed in Table 4, with corresponding results presented in Figure 13. Initial testing employs 10 mm square steel plates as local support elements, with their placement relative to the formed part illustrated in Figure 13a,b. Observations reveal that the local support group, a, positioned adjacent to the deep cavity area of the sharp corners, effectively eliminates wrinkling defects in the outer flange areas, while significant wrinkling persists in nearby deep cavity areas. Here, the redundant material is squeezed to the suspended area of the sharp corner during the formation process. Conversely, the local support group, b, situated entirely within the flange areas outside the sharp corners, restricts horizontal material flow, causing wrinkles to develop vertically rather than horizontally, as observed in unsupported conditions.

The third and fourth experimental configurations employ locally conformal 10 mm steel plate supports, as depicted in Figure 13c,d. The third test series demonstrate minimal wrinkling in the outer flange areas, with only slight wrinkling observed in the sharp corner areas and adjacent deep cavity flange sections. The fourth test series yield results that are comparable to those for group b, exhibiting vertically oriented wrinkles. In addition, it can be concluded that neither increased support rigidity nor elevated compressive stresses of the material can fully eliminate wrinkling defects in the sharp corner areas through altering the local support geometry and placement. Group e implements a combination of 5 mm polyurethane rubber that is conformal to the sharp corners and 5 mm local steel plate flange supports, while 5 mm local polyurethane rubber will replace the steel plate in group f. The results indicate that these configurations significantly reduce wrinkling and effectively control the deformation of material flow in the outer flange regions, though the defects have not been fully eliminated. Ultimately, through optimized thickness and positioning of the local support materials, wrinkle-free sharp corner formation is attained using a 1 mm conformal steel plate with 9 mm local polyurethane rubber, as shown in Figure 13g.

**Figure 14:**
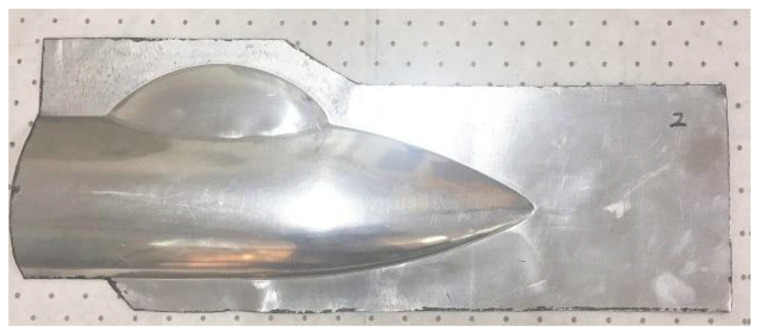
The best-quality fairing part after wrinkling control optimization.

From the experimental results, the conclusion can be drawn that the maximum wrinkle length in the sharp corner area decreases and the wrinkling defect improves with the addition of local support materials. This phenomenon proves that wrinkling is generated due to fiber overlap in that region, and that the placement of local support can alter the compressive stress to some extent. In addition, when the hardness of the support materal is relatively high, like for steel, the material at the support site is rarely deformed, and the flow of the surrounding material may cause other forms of wrinkles. When the material hardness decreases, as with rubber, the wrinkling defect improves as the wrinkle length decreases, although it cannot be completely eliminated. The greatest optimization with no wrinkles is achieved through the combination of a 1 mm conformal steel plate and 9 mm local polyurethane rubber in the sharp corners. This method can be used to improve the flow and deformation of material at local regions of composite laminates. After optimizating the process parameters and local support materials, the best-quality fairing part after hydroforming was identified, as presented in Figure 14.

## 4. Conclusions

In this work, the hydroforming process of an aircraft fairing part composed of fiber metal laminates has been introduced. Wrinkle formation at different locations in two types of lay-up, namely Glare 3/2 and Glare 2/1, was analyzed and compared with wrinkle formation in a single layer of aluminum sheet. A fairing part without wrinkling defects can be obtained through the optimization of process parameters and local support materials. The maximum wrinkle length and forming depth, material flow, and displacement distribution of uncured FMLs during the hydroforming process are discussed after combining both simulation and experimental results. The following conclusions can be drawn from this research:

(1) The wrinkles occurring on the top and bottom flange areas of the fairing are caused by width reduction due to easy material flow into the cavity, while the reason for sharp corner wrinkling is mainly due to fiber overlap under large amounts of compressive stress. In addition, both Glare 3/2 and Glare 2/1 exhibit distinct wrinkling patterns since the neutral layer material of the laminate differs.

(2) The optimization of the process parameters significantly improves wrinkle control in the side flange area. Increasing the blank holder force can greatly reduce wrinkle length, while a higher cavity pressure is required to fill the main shape. Both the experimental and numerical results reveal that the optimal blank holder force during the hydroforming process is 2200 kN for Glare 3/2 and Glare 2/1 laminates; however, the optimal cavity pressure is 28 MPa and 25 MPa for each laminate, respectively.

(3) The addition of local support materials in the sharp corner region helps alter the compressive stress state of the fiber layer, which eliminates wrinkling defects. High-hardness materials like steel are rarely deformed at the support site, resulting in only a small reduction in wrinkle length, whereas low-hardness rubber facilities the easy flow of surrounding material and decreases the possibility of fiber overlap. Complete removal of wrinkles in the sharp corner areas is achieved through the addition of a combination of a 1 mm conformal steel plate and 9 mm local polyurethane rubber in the appropriate position.

## Figures and Tables

**Table 1:** Material lay-up configurations for the laminate hydroforming process.

Type	Materials	Lay-Ups	Total Thickness/mm	
1	Aluminum alloy	Single Layer	0.3	
2	Aluminum alloy + glass fiber prepreg	Glare 2/1	0.8	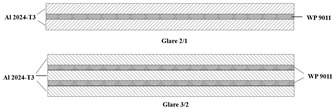
3	Aluminum alloy + glass fiber prepreg	Glare 3/2	1.3	

**Table 2:** Design of experimental parameters for the hydroforming test.

Material Type	Blank Holder Force ($F_{N}-kN$)	Cavity Pressure (P−MPa)
Al 2024-T3-0.3	1500 1700	20 22
Glare 2/1-0.3	2000 2200	25 28
Glare 3/2-0.3	2500	30

**Table 3:** Interface cohesive element parameters and definitions [30].

Definition	Value
Density (kg/m^3^)	ρ=1150
Initial penalty stiffness (MPa/m)	$K_{nn}=K_{ss}=K_{tt}=10^{8}$
Damage initiation strength (MPa)	$N_{0}=\sigma_{peel}=30;\;S_{0}=T_{0}=\sigma_{shear}=60$
Fracture toughness (N/m)	$G_{IC}=260;\;G_{IIC}=G_{IIIC}=1020$
Mixed-mode fit parameter	η=1.45

**Table 4:** Different forms of local support and their wrinkle control effects.

Group	Local Support Material	Maximum Wrinkle Length (mm)	Control Effect
0	No	22.8	Severe wrinkles
a	10 mm square steel plate	14.2	Severe wrinkles
b	10 mm square steel plate	16.5	Severe wrinkles
c	10 mm conformal steel plate	9.7	Slight wrinkles
d	10 mm conformal steel plate	12.4	Severe wrinkles
e	5 mm conformal polyurethane rubber with 5 mm local steel plate	8.8	Slight wrinkles
f	5 mm conformal polyurethane rubber with 5 mm local polyurethane rubber	6.5	Slight wrinkles
g	1 mm conformal steel plate with 9 mm local polyurethane rubber	0.3	No wrinkles

## Data Availability

The original contributions presented in this study are included in the article. Further inquiries can be directed to the corresponding author.
